# Nicotine pouches: A wolf in sheep's clothing

**DOI:** 10.18332/tpc/211847

**Published:** 2026-02-04

**Authors:** Luis F. Gonzalez, Theodore C. Friedman

**Affiliations:** 1 Department of Psychology, California State University San Bernardino, San Bernardino, United States; 2 Department of Internal Medicine, Charles R. Drew University of Medicine and Science, Los Angeles, United States

**Keywords:** regulation, harm reduction, nicotine addiction, tobacco-free, nicotine pouches

## Abstract

Nicotine pouches are a fast-growing class of tobacco-free nicotine products, marketed for oral absorption and often promoted as cleaner alternatives to traditional tobacco. Regulatory efforts to ban flavors such as menthol have been inconsistent, with the US withdrawal of a proposed menthol ban in 2025 intensifying policy instability and raising concerns over youth use. The aim of this monitoring letter is to examine the development of nicotine pouch markets, regulatory responses, and the need for independent research to guide future policy. Sales of nicotine pouches increased dramatically from their US introduction in 2016, with youth uptake rising alongside aggressive marketing and flavored product availability. ZYN commands nearly 68.7% of the youth market, supported by social media campaigns and influencer endorsement. Despite the FDA's 2025 authorization of select ZYN products, most brands remain unregulated. Research primarily sponsored by industry suggests harm reduction, yet independent studies have found carcinogens and toxic metals in some products, and evidence gaps persist regarding health risks and long-term outcomes. International regulation varies, with France instituting a nationwide ban in 2025, contrasting sharply with the US approach. Nicotine pouches continue to expand in both market reach and youth appeal, driven by flavor options and harm-reduction narratives. Regulatory and scientific oversight remain fragmented, echoing previous challenges faced with e-cigarettes. Coordinated policy action and independent research are urgently needed to prevent a repeat of prior youth and public health concerns.

## INTRODUCTION

Nicotine pouches have emerged as a rapidly growing category of smokeless, tobacco-free nicotine product designed for oral absorption through the mucosa^1,2^. In 2022, both the World Health Organization (WHO) and the US Food and Drug Administration (FDA) recommended banning menthol flavoring for cigarettes, but enforcement was inconsistent^3^. However, the current US administration withdrew the proposed menthol ban in January 2025^4^. This reversal underscores the instability of tobacco regulatory policy and its potential impact on youth-oriented (12–24 years) nicotine markets^5^.

Marketed as ‘tobacco-free, cleaner, and intense’ alternatives^6^. Nicotine pouches generally contain nicotine salts, flavoring agents and sweeteners, and are placed between the gum and lip for absorption^1,2,7^. Some products deliver up to 50 mg of nicotine per pouch^8^, and can be used discreetly without batteries or devices^7^.

Leading manufacturers include Swedish Match (ZYN, now part of Philip Morris International), British American Tobacco (Velo), Kretek International (DRYFY), Japan Tobacco International (Nordic Spirit), and Altria (On!)^2,7,9-13^. Despite this brand diversity, youth consumption remains concentrated around a few products, by which ZYN dominates with 68.7% market share among youth, followed by On! (14.2%), Rogue (13.6%), Velo (10.7%) and Juice Head ZTN (9.8%)^14,15^.

Biomarker analyses suggest nicotine pouches may reduce exposure to certain toxicant compared to combustible tobacco, although nicotine absorption remains similar^16,17^. Manufacturers frame these results as evidence of harm reduction, yet independent verification is limited. Since their US market introduction in 2016, nicotine pouch sales have grown dramatically. ZYN emerged as the market leader by 2023^11^, even as cigarette sales declined^6,10,18-20^. By 2024, the U.S. National Youth Tobacco Survey identified nicotine pouches as the second most prevalent tobacco-related product among youth, with 1.8% reporting use^14^. Global market projections estimate revenues near $23 billion by 2030^21^.

The aim of this monitoring letter is to examine the development of nicotine pouch markets, regulatory responses, and the need for independent research to guide future policy.

## COMMENTARY

### Market trends, development and youth uptake

**Figure 1:**
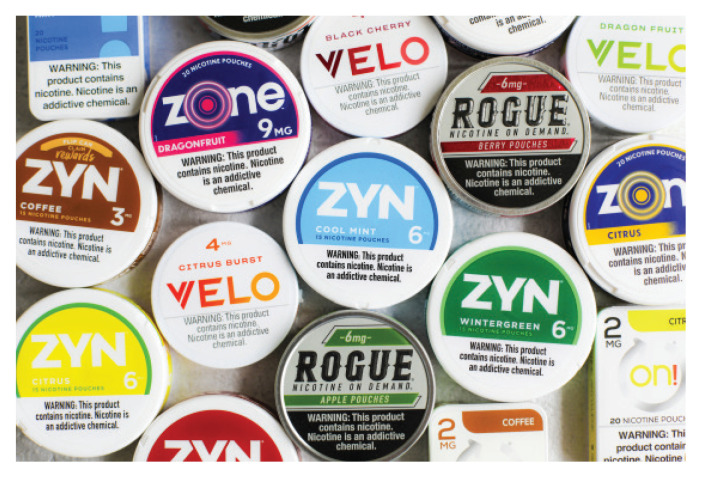
Popular nicotine pouch brands in New York on 9 July 2025. The image shows leading smokeless nicotine pouch products available in the United States: including ZYN, VELO, Rogue, and Zone. Documented at 12:21 PM PST. This selection reflects market trends and top options for early 2025

Nicotine pouch sales surged from 163000 units in 2016 to over 45 million by mid-2020^11^, reflecting their distinctness from traditional smokeless tobacco through the use of pharmaceutical-grade nicotine instead of leaf tobacco^22^. Youth use (ages 12–24 years) rose from 3.0% to 5.4%, outpacing adults use increases (2.9% to 3.3%)^12,23,24^. Between 2019 and 2021, the tobacco industry spent approximately $1.5 billion on marketing; emphasizing youth appealing flavors (fruit, mint, coffee) (Figure 1), discreet use and social media outreach especially on TikTok, where ZYN gained major traction^7,10,12,16,22-26^. Influencers such as Tucker Carlson have promoted ZYN as a safer alternative, though clinical evidence remains limited^25,27^. Among youth users, 85.6% favor flavored products with most preferring nicotine strengths of 6–10 mg^26,28^. While youth prevalence remains lower for nicotine pouches than for e-cigarettes (8.7% vs 19.7%)^29^, awareness and curiosity continue to rise^1,18,19,29^.

In January 2025, ZYN became the first nicotine pouch brand to receive FDA marketing authorization, legally permitting the sale of specific products in the US^30^. The FDA determined these pouches met the public health standard required by the 2009 Family Smoking Prevention and Tobacco Control Act^31^, which weighs risks and benefits to the population. Evidence showed nicotine pouches contain significantly fewer harmful substances than cigarettes and most smokeless tobacco products, such as moist snuff and snus, thereby posing a lower risk of cancer and other serious health conditions^32^.

Swedish Match presented non peer-reviewed, company-funded studies indicating that 24% of adults who used cigarettes and/or smokeless tobacco fully switched to nicotine pouches, and that dual use with cigarettes declined from 42% to 15% over five to six months^32^. Based on these findings, the FDA concluded nicotine pouches may benefit adult smokers or smokeless tobacco users by reducing harm, though they remain unsafe and are not ‘FDA approved’. No tobacco product is safe; youth should not use them, and non-users should not start.

We recommend the need for independent, peer-reviewed research to assess safety and efficacy, and recommend comparative studies of e-cigarettes versus nicotine pouches for smoking cessation.

### Regulation and oversight

The regulatory landscape for nicotine pouches continues to evolve. The FDA requires premarket applications for all new nicotine pouches; yet as of 2025, only ZYN products have received authorization, while many unauthorized brands continue to proliferate through retail and social media platforms^10,11,21,22,29,33^. Regulatory gaps persist because oral nicotine pouches do not fit neatly withing existing tobacco regulations^22^. The challenging enforcement history of e-cigarettes remains because restrictions only took effect nearly a decade after initial regulator intent and illustrates how challenges persist in enabling youth-oriented market expansion^21^. By mid-2024, 34 e-cigarette products had marketing authorization, though unauthorized products still dominated the market^21^. Similarly, gaps in nicotine pouch regulation could result in outcomes similar to past public health challenges. Timely FDA action may help guide market development and strengthen youth protection measures.

Internationally, regulation varies widely. In 2025, France issued a nationwide decree banning the production, distribution, and the use of oral nicotine pouches^34^. This signals strong recognition of their emerging risk and contrasts sharply with US regulatory strategies^34^.

### Research gaps

Most available evidence on nicotine pouches originate from industry-funded research, such as Swedish Match-sponsored reports suggesting smoker can fully transition from cigarettes to pouches^32^. However, these claims lack independent validation, and non-industry studies have detected carcinogenic tobacco-specific nitrosamines in some products^20,32^. Independent analysis has also identified traces of chromium and formaldehyde^35,36^. Available research primarily examines acute exposure^37^, leaving critical evidence gaps regarding:

1. The absence of animal models for studying pouch exposure.
2. Limited comparative data between pouch use and e-cigarettes.
3. Scarce research on long-term health outcomes.

Future research should prioritize independent, peer-reviewed studies. Regulatory policy should mandate transparent product labeling, restrict youth-oriented marketing and coordinate international cooperation to avoid regulatory loopholes.

## CONCLUSION

Since their 2016 debut, nicotine pouches have expanded rapidly, particularly among youth. Although ZYN gained FDA marketing authorization in 2025; most products remain unregulated, and flavored formulation continues to drive popularity under harm-reduction marketing narratives. France’s 2025 nationwide decree underscores growing concern over nicotine pouches as a global public health issue. Sustained monitoring, prompt regulatory intervention, and independent research are essential to prevent youth uptake patterns similar to those previously observed with e-cigarettes.

## Data Availability

Data sharing is not applicable to this article as no new data were created.
